# Spatial distribution of suicide in Queensland, Australia

**DOI:** 10.1186/1471-244X-10-106

**Published:** 2010-12-07

**Authors:** Xin Qi, Shilu Tong, Wenbiao Hu

**Affiliations:** 1 School of Public Health, and Institute of Health and Biomedical Innovation, Queensland University of Technology, Kelvin Grove, Queensland 4059, Australia; 2 School of Population Health, University of Queensland, Herston, Queensland 4006, Australia

## Abstract

**Background:**

There has been a lack of investigation into the spatial distribution and clustering of suicide in Australia, where the population density is lower than many countries and varies dramatically among urban, rural and remote areas. This study aims to examine the spatial distribution of suicide at a Local Governmental Area (LGA) level and identify the LGAs with a high relative risk of suicide in Queensland, Australia, using geographical information system (GIS) techniques.

**Methods:**

Data on suicide and demographic variables in each LGA between 1999 and 2003 were acquired from the Australian Bureau of Statistics. An age standardised mortality (ASM) rate for suicide was calculated at the LGA level. GIS techniques were used to examine the geographical difference of suicide across different areas.

**Results:**

Far north and north-eastern Queensland (i.e., Cook and Mornington Shires) had the highest suicide incidence in both genders, while the south-western areas (i.e., Barcoo and Bauhinia Shires) had the lowest incidence in both genders. In different age groups (≤24 years, 25 to 44 years, 45 to 64 years, and ≥65 years), ASM rates of suicide varied with gender at the LGA level. Mornington and six other LGAs with low socioeconomic status in the upper Southeast had significant spatial clusters of high suicide risk.

**Conclusions:**

There was a notable difference in ASM rates of suicide at the LGA level in Queensland. Some LGAs had significant spatial clusters of high suicide risk. The determinants of the geographical difference of suicide should be addressed in future research.

## Background

Suicide is a major cause of death around the world with about 877,000 suicide deaths each year globally [1]. The World Health Organization has predicted that the suicide rate will steadily increase into the future [2].

In Australia, the trend of suicide has fluctuated over the 20th Century and early 21^st ^Century [3,4]. In recent years, there have been over 2000 suicide cases recorded annually in Australia (ABS 2003, 2004) [4], with males accounting for the majority of these suicides. A number of studies have explored the distribution of suicide in different states in Australia [5-9].

Some Australian and international studies have applied spatial analysis to assess the geographical difference in suicide incidence [10-15]. Our previous study analysed the spatiotemporal association between socio-environmental factors (climate, socioeconomic and demographic factors) and suicide in Queensland, Australia [13]. Some other studies also explored the spatial variation of suicide in Queensland (14-15). At an international level, several studies have explored the geographic distribution of diseases using spatial cluster analysis [12,16-18], identifying clustering in several diseases, including suicide [12]. Spatial cluster analysis is a vital tool because it helps to find the clusters of any disease with high or low relative risk. Each cluster consists of several geographic units linked together, and has a small proportion of the population (e.g., less than 25%) of that in the whole study area. All these studies on spatial cluster analysis were implemented in countries (regions) with much higher population density than that of Australia, which varied among urban, rural and remote areas. The patterns of suicide may differ between Australia and other countries. Thus it is important to examine the spatial clusters of suicide in Australia, to improve current suicide control and prevention strategies.

This study aimed to examine the spatial distribution of suicide at a LGA level and identify the LGAs with a high relative risk of suicide in Queensland, Australia, using geographical information system (GIS) techniques. Queensland is the second largest state in Australia, in terms of areas and lies in the northeast of the country with an area about 1.73 million km^2 ^and a total population of 4.41 million in June 2009. Southeast Queensland (SEQ) covers less than 1.3% of the total area, but had 65.4% of total population while other places have much lower levels of population density than that of the SEQ. The economy in Queensland has increased more rapidly than that of other areas in Australia since 1992, except for the financial year 1995-1996 [19]. Mining, financial services and tourism are the major industries in Queensland.

## Methods

### Data sources

Suicide data were obtained from Australian Bureau of Statistics (ABS), including gender, age, year and month of suicide (January 1999 to December 2003), country of birth and code of Statistical Local Area. The year 2003 is the cut-off in this study because this dataset was obtained a few years ago. Currently ABS does not accept any application for accessing the detailed mortality data as it is reviewing its services process. This study involved 2,445 suicide deaths from 1999 to 2003, with 1957 males and 488 females (male/female ratio: 4.01). As it is time-consuming and computation intensive to calculate the age-standardised mortality (ASM) rates at a Statistical Local Area (SLA) level, we used the aggregated data to examine the feasibility of linking different sources of data in this study. The ethical application for this study was approved by University Human Research Ethics Committee, Queensland University of Technology (Approval Number: 1000000220).

According to ABS, there were 452 SLAs in Queensland in 2001. In Queensland, there were 489 suicides on average each year from 1999 to 2003 and each SLA had only about 1 suicide every year on average (range: 0 to14) so it is difficult to detect the spatial pattern of suicide at a SLA level. Previous research on suicide in England and Wales discussed a similar problem [10]. Due to the low total suicide rates within each SLA, the larger geographic boundary area, Local Governmental Area (LGA), was used to detect areas of suicide relative risk or clustering. Urban LGAs contain two or more SLAs (e.g., Brisbane City had 163 SLAs in 2001), and in rural and remote areas that make up the majority of Queensland territory, each LGA is also an SLA. The LGA information, including name, code and area (km^2^), was collected from Census Data (CDATA) 2001, a database developed by ABS which provides information of 2001 Australian Census of Population and Housing, digital statistical boundaries and base maps. There were 125 LGAs in Queensland in 2001. All suicide data were then compiled and linked at the LGA level. The Australian Standard Geographical Classification ASGC (1999-2003) was applied as a reference to combine the SLAs into LGAs. MapInfo 9.0 was used as a platform to perform the data linkage, transfer and spatial display.

Population data in total, by gender and age groups (i.e., ≤24-years for youth and adolescents, 25 to 44-years for young adults, 45 to 64-years for middle-aged adults, and ≥65-years for elderly) at a LGA level, were also collected from CDATA.

### Data analysis

A series of GIS and statistical methods were used to analyse these data. MapInfo (including Vertical Mapper) was used to explore spatial patterns of suicide by gender, age and LGA. SaTSCAN was applied to analyse the spatial clusters of suicide across LGAs.

In order to examine the spatial patterns of suicide, ASM rates by gender for each LGA were calculated by a direct method. The data on the population structure by age and gender at a LGA level in Queensland were obtained from ABS. The equation for calculating ASM is as follows:

$$ASM=\frac{\sum N_{i}p_{i}}{N},$$

where *N_i _*is the standard population size in each LGA by age and gender, *p_i _*represents the death rate of each LGA by age and gender, and *N *is the total population of Queensland. Four steps were used to calculate the ASM for each LGA in this study:

1. Obtain the total number of suicides in the LGA by age and gender.

2. Calculate the gender age-specific rates of suicide deaths per 100,000 for each LGA.

3. Calculate the expected number of deaths (*N_i_p_i_*) by age and gender for each LGA.

4. Sum the expected number of deaths and divide by the total population of Queensland to get ASM per 100,000 for each LGA.

Statistical analyses, including both descriptive and spatial analysis approaches, were performed to examine the spatial distribution of suicide by LGA and gender. Descriptive analysis was used to explore the characteristics of each variable. Spatial analysis was performed to view the spatial distribution of suicide ASM rates by gender and age, using GIS and mapping approaches. The MapInfo Professional (version 8.5) and Statistical Package for the Social Sciences (SPSS, version 16.0) were used for data management and analysis [20,21].

Spatial cluster analysis was implemented to detect whether the suicide cases were randomly distributed and to explore the spatial clusters of suicide. In the spatial cluster analysis, the suicide relative risk (RR) of each LGA was calculated using a Poisson model, and the mean RR of each cluster (including one or more LGAs) was also computed with the SaTSCAN (version 8.0) [22]. The annual average mortality (total and by gender) of the whole state (1999-2003) was defined as the reference for the RR in each LGA. To identify whether selection of population size influences the size of clusters, the spatial clusters were defined to cover less than 50%, 25% and 10% of total population respectively, including both most likely cluster(s) and secondary likely cluster(s). The longitude and latitude of the centroids in each LGA were used in the analysis. The most likely and secondary likely clusters were indicated through the likelihood ratio test and indicated as circular windows, to test the hypothesis that these areas had an elevated risk compared to other areas.

## Results

Table 1 indicates the distribution of suicides by age and gender. Most of the suicide cases were aged between 25 and 64 years, with male suicides accounting for approximately 80% of all deaths.

**Table 1:** Suicide by gender and age in Queensland (1999-2003)

Age	Males	Females	Total
24 & below	321	74	395
25-44	917	237	1154
45-64	482	126	608
65 & over	237	51	288
Total	1957	488	2445

Table 2 reveals that suicide mortality rates, particularly male ASM, varied substantially across LGAs. For example, Brisbane City had an area of 1,327 km^2 ^with a population of 888,499 (2001 census data) and 565 suicide cases recorded. The Diamantina Shire covers 94,832 km^2^; it had a population of only 448 persons (2001 census data) and no suicides were recorded between 1999 and 2003. Therefore, population density was not regarded as an indicator of suicide rates.

**Table 2:** Suicide mortality rates by gender (N = 125)

				Percentiles			
					
	Mean	Std. Deviation	Minimum	**25**^**th**^	**50**^**th**^	**75**^**th**^	Maximum
Mortality (per 100,000)	17.12	27.876	0.00	8.40	13.03	19.08	296.30
Male ASM (per 100,000)*	28.11	46.873	0.00	14.11	20.72	30.65	492.81
Female ASM (per 100,000)*	5.50	11.023	0.00	0.00	1.75	6.70	87.34

*ASM: age standardised mortality rate

**Figure 1:**
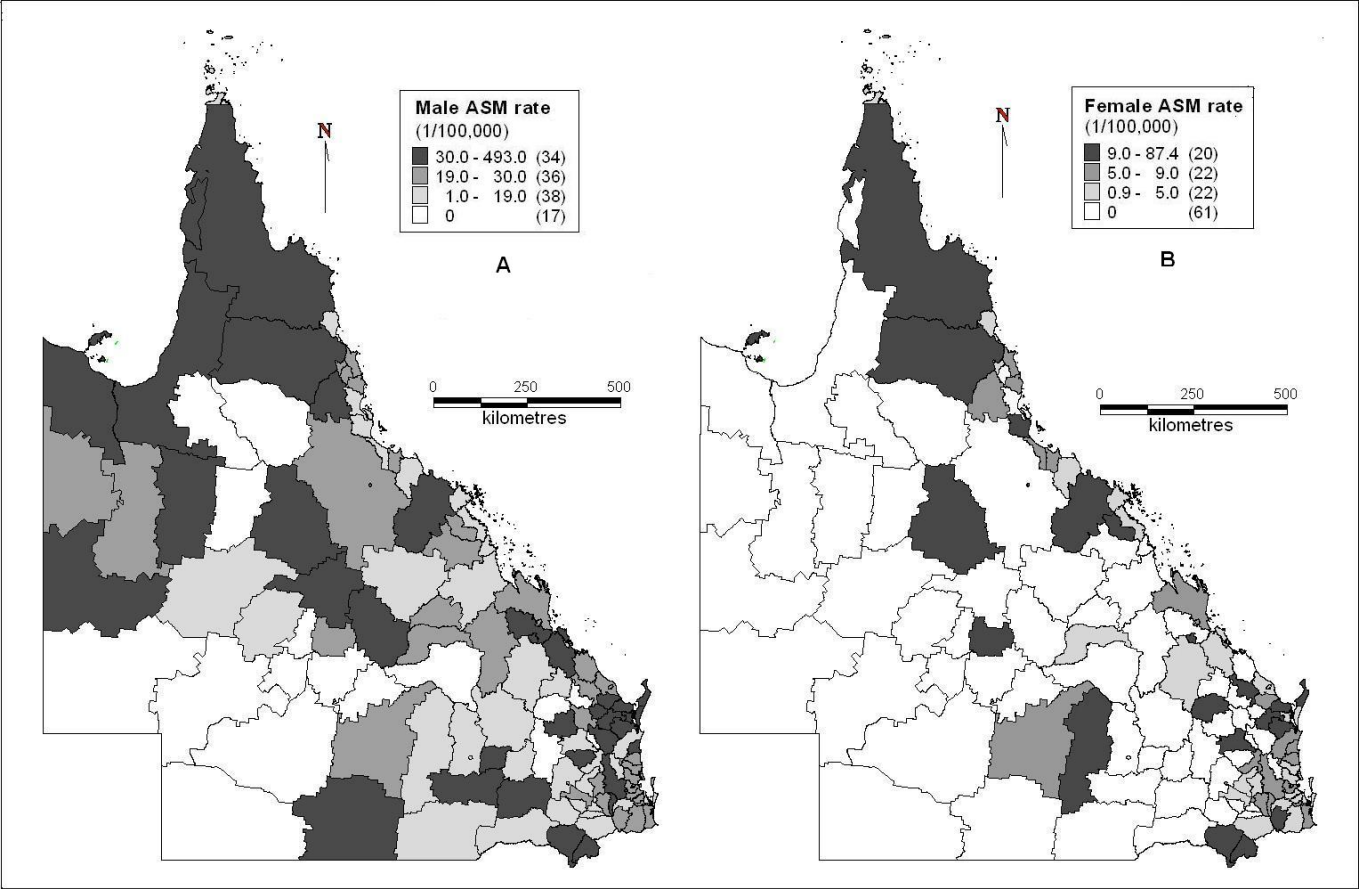
**Suicide age standardised mortality rates (A: male; B: female) in Queensland (1999-2003)**.

Figure 1A shows the map of average male suicide ASM rates at the LGA level in Queensland. It indicates that central Queensland, far north (part of Peninsula of Cape York), north-western areas (coastal areas of Gulf of Carpentaria), part of western, part of southern, south-eastern coastal and eastern areas had higher suicide ASM rates, while northern-central, south-western, southern and south-eastern inland areas had lower rates.

Figure 1B shows female suicide ASM rates at the LGA level. Part of central, eastern and southern coastal areas had higher female suicide ASM rates compared with other areas. However, almost half of 125 LGAs in Queensland had no suicides recorded during 1999 and 2003.

**Figure 2:**
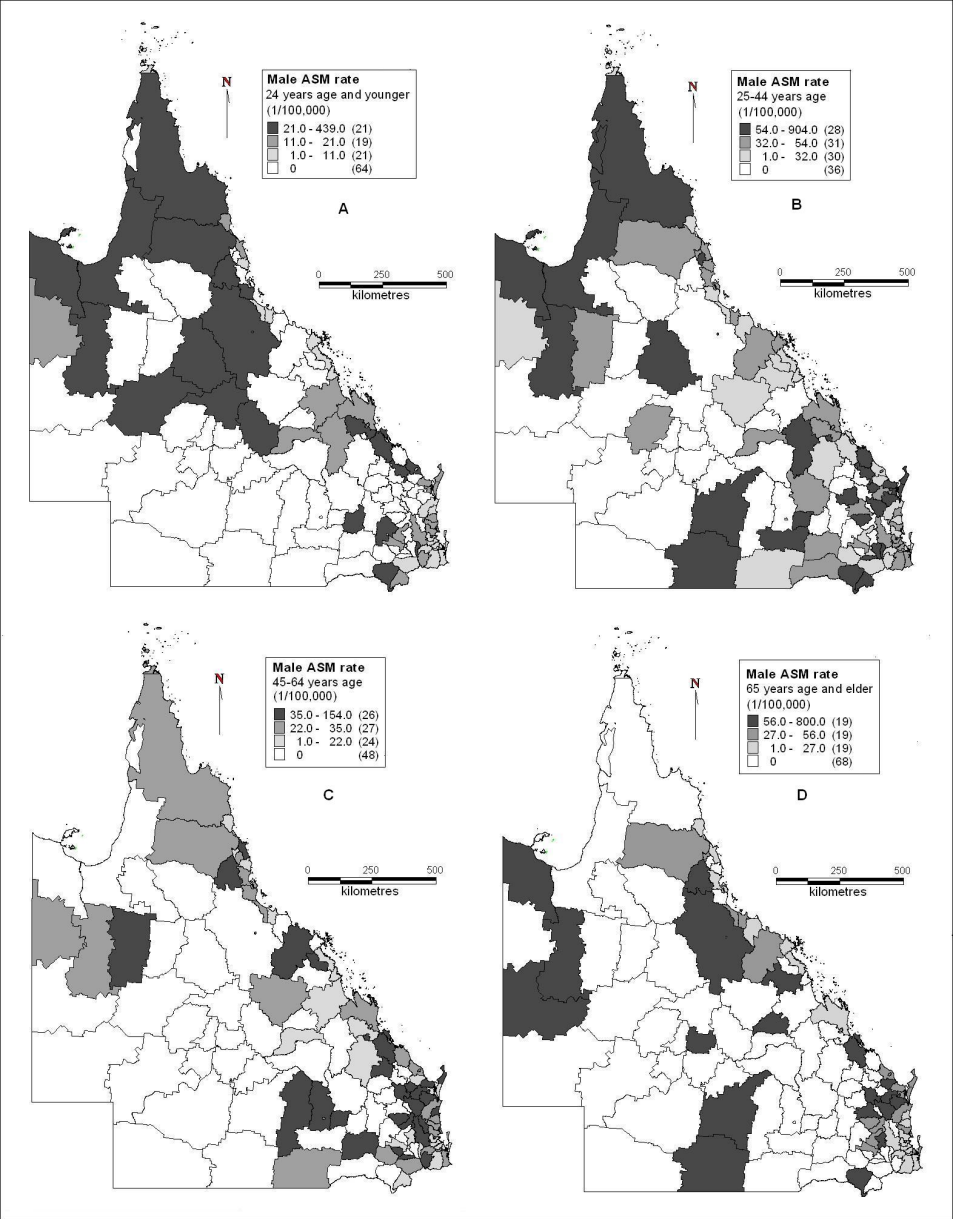
**Male suicide age standardised mortality rates in Queensland (A: 24 years and younger; B: 25-44 years age; C: 45-64 years age; D: 65 years age and above)**.

Figure 2A show the spatial distribution of male suicide ASM rates in different age groups. Figure 2A indicates that among youths and adolescents, the far north, northwestern, part of central and north, central coast and southeastern areas had higher suicide ASM rates, while central inland, northern coast, south and southwest areas had lower rates during the study period. Among young adults, part of far north, northwestern, part of central and southern areas had higher suicide ASM rates, while part of north, southwestern and central south areas had low suicide rates (Figure 2B). Figure 2C shows that among middle-aged adults, part of the far north, west, central south and southeast areas had higher suicide ASM rates, while north, northwest, central inland and southwestern areas had lower rates. Among the elderly, the part of northwest, west, north, part of the south, central and part of the southeastern areas had higher suicide ASM rates, while the far north, central inland, southwest, and part of the south areas had lower rates during the study period (Figure 2D).

**Figure 3:**
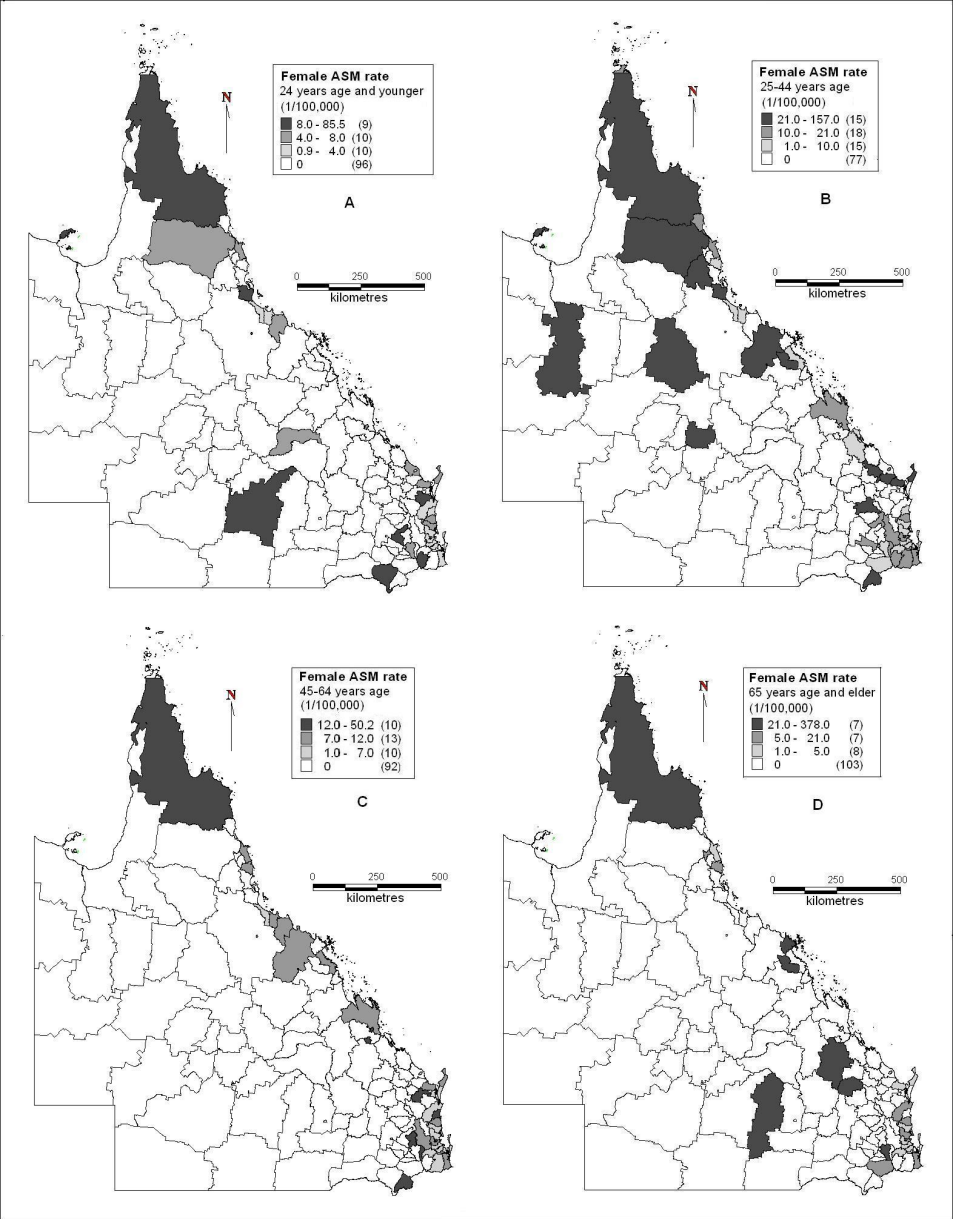
**Female suicide age standardised mortality rates in Queensland (A: 24 years and younger; B: 25-44 years age; C: 45-64 years age; D: 65 years age and above)**.

Figure 3A - D revealed the spatial distribution of female suicide ASM rates in different age groups. Among youths and adolescents, the far north, part of the north coast and northwest, part of the central inland and southeast areas had higher suicide ASM rates, while over 76% of all LGAs had no suicides recorded during 1999 to 2003 (Figure 3A). Figure 3B shows that among young adults, there were higher suicide ASM rates in the far north, part of the northwest, north coast, part of inland and southeast areas, while lower suicide rates (or no suicides recorded) were observed in most of north, central, south and southwest areas. For middle aged adults, the far north and part of the south east areas had higher suicide ASM rates, while 75% of all the LGAs had no suicides recorded (Figure 3C). Among the elderly, far north, part of north and central coast, part of central south inland and southeast areas had higher suicide ASM rates, while over 82% of all LGAs had no suicides recorded (Figure 3D).

**Figure 4:**
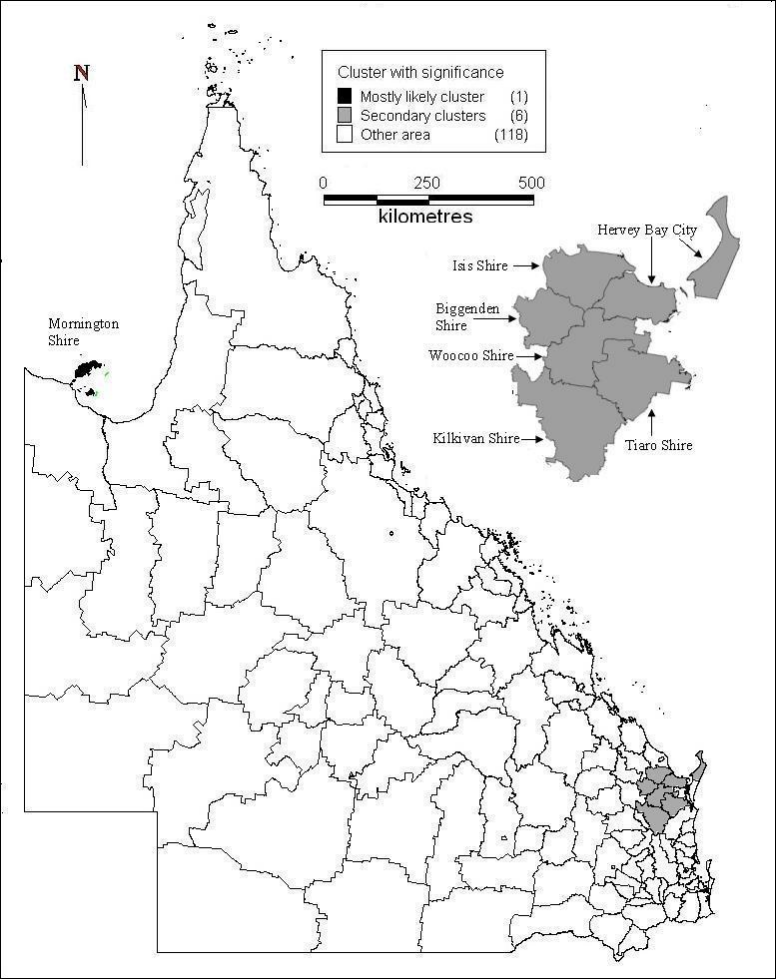
**Clusters of high suicide (total and male) risk area with significance in Queensland (cluster enlarged)**.

In the spatial cluster analysis, suicide was not randomly distributed. Figure 4 indicates the cluster areas of high suicide risk (both total and male) in the whole state. Mornington Shine in the northwest was the mostly likely cluster, but the neighbouring LGAs (e.g., Burke and Carpentaria Shires) did not demonstrate clustering, although these areas had high suicide ASM. The secondary likely cluster contains six LGAs in upper Southeast Queensland (SEQ).

These clusters contained 1.77 per cent of the total population in the whole study area with 3.8 per cent of total suicides. Table 3 shows the details of clusters for total and male suicides. Different cluster sizes (e.g., radius of 200 km and 400 km) and population (less than 10%, 25% and 50% of total) were tested and no apparent difference in the results was found from various selections. The clusters of low risk areas for male suicide were also tested but no cluster was discovered. For female suicide, no cluster area of high and low suicide risk was identified during the study period.

**Table 3:** Spatial clusters of suicide in Queensland

	Mostly likely cluster	Secondary likely cluster
**LGA names**	Mornington (S)	Biggenden (S), Isis (S), Hervey Bay (C), Kilkivan (S), Tiaro (S), Woocoo (S)
**Cluster radius (km)**	0	57.01
**Area (km^2^)**	1231.25	12830.13
**Population**	945 (T), 487(M)	64,054 (T), 31,839 (M)
**Number of cases**	14 (T), 12 (M)	79 (T), 64(M)
**Expected cases**	0.63 (T), 0.53 (M)	42.87 (T), 34.49 (M)
**Relative risk**	22.26 (T), 22.88 (M)	1.87 (T), 1.88 (M)
**P value**	0.001(T & M)	0.001 (T), 0.005 (M)

*S: Shire; C: City; T: Total; M: Male.

## Discussion

This study examined the spatial distribution of suicide in Queensland by gender and age. Male suicides accounted for 80% of total suicide cases and 47% of total suicides were young adults. In general, the maps of this study show that part of far north and north, northwest, some of west, central and east areas had higher male suicide ASM rates, while southwest and some of central areas had no male suicides recorded. Far north Queensland, part of the northwest, coastal and central areas had higher female suicide ASM rates, but almost half of the LGAs had no female suicide cases recorded. Suicide mortality also varied between LGAs among both genders in different age groups.

SEQ covers less than 1.3% of total area of the state, but accounts for 65.4% of the total population and 62.4% of total suicides in Queensland [23]. In SEQ, the suicide ASM rates were relatively similar across LGAs, except for female youths and adolescents. Thus it is difficult to find the cluster of high risk suicides in SEQ. The number of LGAs with female elderly suicides was the least compared for numbers of LGAs with suicides in other age and gender groups. In LGAs with a low population (i.e.., less than 2000), the ASM rates were often higher than other LGAs if suicides occurred. For example, Mornington Shire in northwest Queensland had a population of 945 in 2001, but it had 14 suicide cases in the 5-year study period.

**Figure 5:**
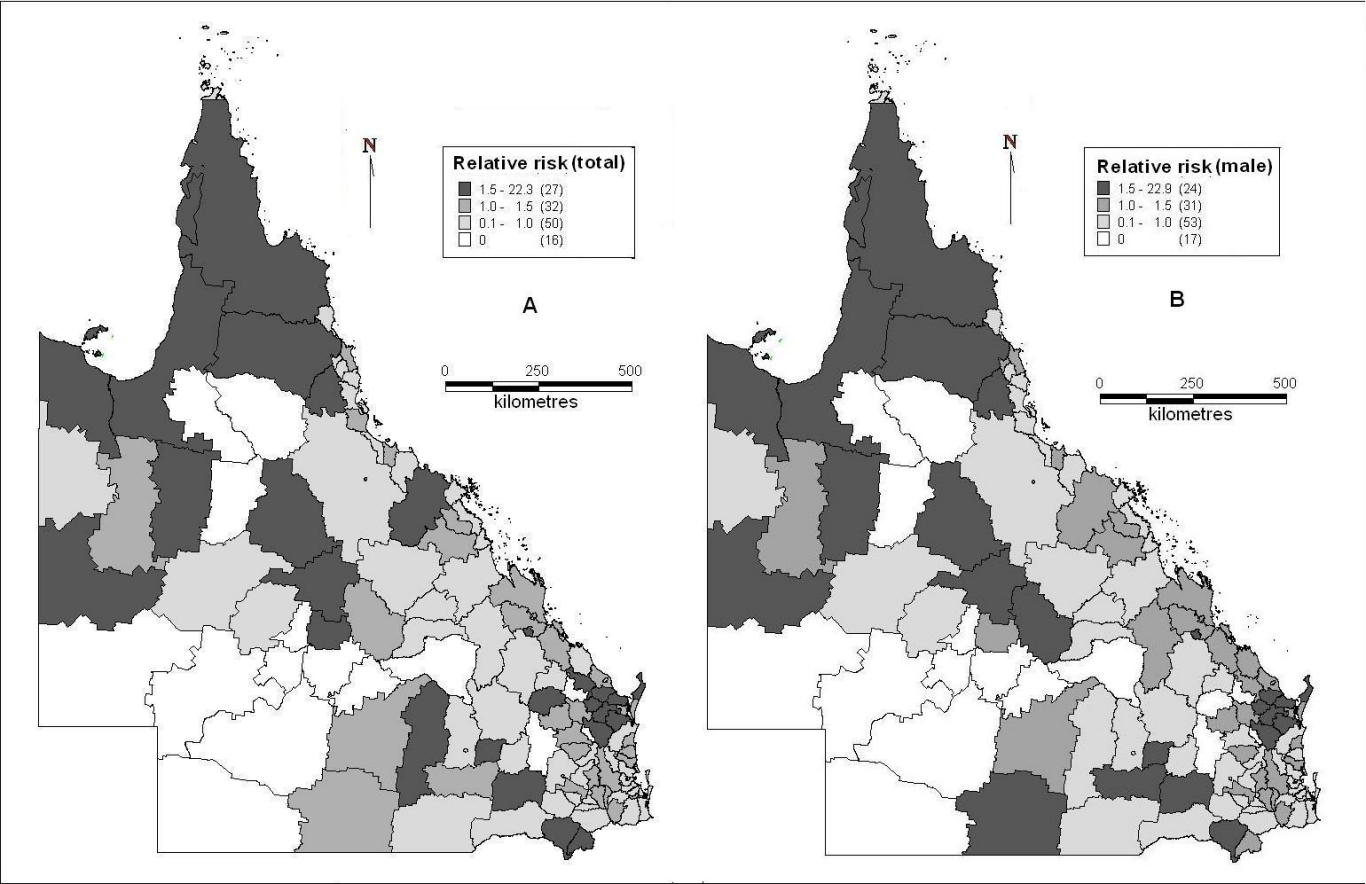
**Suicide relative risk (A: total; B: male) in Queensland**.

The spatial cluster analysis discovered significant clusters of Mornington Shire in the northwest and six other LGAs in upper SEQ. Seven LGAs in the far north (Aurukun, Burke, Carpentaria, Cook, Herberton, Mareeba and McKinlay Shires) are linked together with RRs, between 1.5 and 5.8 (total and male) in each but not in any cluster (Figure 5). These LGAs cover 19.6 per cent of the whole study area but had only 1.15 per cent of the total population and 2.45 per cent of total suicides, which means a very low population density but higher suicide rates compared with the average suicide rate in the whole state. This may be due to social isolation and the lack of mental health services available in these areas [17,24]. The SaTScan has the maximum limit in controlling the radius and population of clusters; therefore LGAs in the far north mentioned above, covering a large proportion of the whole study area, could not be selected as clusters by SaTScan. This may explain the discrepancy between LGAs with a high relative risk of suicide and LGAs with clustering.

Most of the LGAs with higher suicide ASM rates were shires whose populations were predominately composed of Aboriginal and Torres Strait Islander. Some studies in Queensland have indicated that Indigenous areas have higher suicide mortality than other areas [13,25]. Most of the Indigenous areas have low socioeconomic status as well as fewer opportunities to seek mental health care. The Indigenous communities have also been influenced by the rapid social change in Australia. The prevalence of unhealthy behaviours (e. g., excessive alcohol use) and family violence has increased in recent years [26], factors that may have contributed to higher suicidal activities and deaths in Indigenous communities [26]. Other studies in Australia also support these opinions [27-29]. The ABS published the Socio-economic Indexes for Area (SEIFA) at the Statistical Division (SD) and LGA levels, including four indexes: the Index of Relative Socio-economic Advantage and Disadvantage (IRSAD), the Index of Relative Socio-economic Disadvantage (IRSD), the Index of Economic Resources (IER) and the Index of Education and Occupation (IEO) [30]. The higher each variable indicates higher socioeconomic status (SES) in each SD/LGA. Our previous study in Queensland has indicated that LGAs with higher SEIFA usually have lower suicide mortality [13]. In this study, the SD of Wide Bay-Burnett (contains all the LGAs of the cluster in the upper Southeast) had the lowest IRSAD, IER and IEO and second lowest (higher than the Northwest) IRSD among all the 11 SDs in Queensland. At the LGA level, Mornington Shire ranked between the lowest 2^nd ^and 14^th ^among all 125 LGAs in each index of SEIFA. This may contribute to the cluster of high risk of suicide in the whole state. Other studies also show similar results, especially in a long study period (e.g., over 30 years) when suicide prevention strategies were implemented and their effects emerged over such a period [31,32]. A recent study by Large and Nielssen indicated that in Australia, suicide mortality was lower in the decade 1998 to 2007 than that in the decade 1988 to 1997 as the availability of lethal methods of suicide decreased and there was also a sustained period of economic prosperity for most sections of society [33].

### Strengths and limitations

This study has three key strengths. Firstly, it is the first study to examine the spatial distribution of suicide in Queensland at a LGA level using a spatial cluster analysis approach. The spatial cluster analysis can identify clustered areas with high risk of suicide, and this helps researchers to both explore the factors associated with clusters of high risk, and to address the public health implications of these clusters in suicide control and prevention. Secondly, this study explored the spatial pattern of suicide in different gender and age groups, using GIS techniques. Finally, the results of this study may assist in identifying high risk areas of suicide and developing more effective suicide control and prevention strategies.

This study has several limitations. Firstly, the period of collection of data related to suicide is relatively short, so it is difficult to examine long term trends of suicide at the LGA level. Secondly, the demographic data at the LGA level were only based on the 2001 Population Census, so they cannot reflect any changes in demographic features during the whole study period. Thirdly, the information of home address of suicides was not available due to ethical issues. There is a potential for misclassifying some suicide cases into different LGAs if their houses were on boundaries areas, particularly when boundaries changed. The difficulties in accurate suicide data collection and reporting existed due to less specific classification of suicide causes from deaths by ICD Code [34]. Finally, it is difficult to determine whether the spatial clusters were related to events that took place soon within a short space of time, or were evenly spaced over time and location within high risk communities. This issue should be addressed using a spatiotemporal approach.

### Future research and policy recommendations

A few recommendations can be drawn from this study. Firstly, most suicide cases occurred in Brisbane and other cities in SEQ, while the Wide Bay-Burnett had a cluster of high risk areas for suicide. Thus suicide control and prevention programmes should focus on these areas, especially at the high risk clusters and the far north areas. Secondly, further research should be conducted focusing on the areas with high clustering or high relative risks. Factors such as mental health and community issues (e.g. alcohol abuse, domestic violence and social disadvantage) in these areas and their associations with suicide should be studied. Thirdly, socio-environmental factors (e. g., meteorological factors like temperature and rainfall [13,35,36], and socioeconomic factors like income [37] and unemployment [13,38] may have significant impacts on suicide. Agriculture types [39] and natural disasters [40,41] have a socioeconomic impact on rural areas, which may lead to mental health problems and even suicide behaviours. The association between these factors and suicide at a LGA or other geographical areas need to be explored. A spatiotemporal analysis should be implemented in future research to examine how suicide incidence changes over time and space. Finally, the results in current and future research may provide epidemiological evidence for an improvement of the current suicide control and prevention programs.

## Conclusions

In this study, we discovered that suicide ASM varied between LGAs by gender and age. Far north and north-eastern Queensland had the highest suicide incidence for both genders, while the south-western areas had the lowest incidence for both genders. Mornington and other six LGAs with low socioeconomic status in the upper Southeast had significant spatial clusters of high suicide risk. It suggests that public health interventions for suicide should target these high risk areas. These findings may have implications for implementing and improving population-based suicide interventions in Queensland, Australia. This spatial analysis method may also have a wide application in mental health research and practices.

## Abbreviations

ABS: (Australian Bureau of Statistics); ASM: (age-adjusted standardized mortality); GIS: (geographical information system); LGA: (Local Governmental area); RR: (relative risk); SEIFA: (Socioeconomic Indexes for Areas); SD: (Statistical Division); SLA: (statistical local area).

## Competing interests

The authors declare that they have no competing interests.

## Authors' contributions

XQ designed the study, implemented all statistical analyses and drafted the manuscript. ST conceptualised the idea and revised the study protocol, especially the research design and data analysis. WH contributed to statistical analyses and interpretation of the results. All the authors contributed to the preparation of the final manuscript and approved the submission.

## Pre-publication history

The pre-publication history for this paper can be accessed here:

http://www.biomedcentral.com/1471-244X/10/106/prepub
